# Simultaneous Ipsilateral Floating Hip and Knee: A Complex Combination and Difficult Surgical Challenge

**DOI:** 10.1155/2020/9197872

**Published:** 2020-02-10

**Authors:** Abdellatif Benabbouha, Mostapha Boussouga, Salaheddine Fjouji, Adil Lamkhanter, Abdeloihab Jaafar

**Affiliations:** ^1^Department of Orthopedic Surgery and Traumatology, Military Hospital Mohammed V (HMIMV), University Mohammed V, BP 10100 Rabat, Morocco; ^2^Department of Anaesthesiology, Military Hospital Mohammed V (HMIMV), University Mohammed V, BP 10100 Rabat, Morocco

## Abstract

Simultaneous ipsilateral floating hip and floating knee are extremely rare. To the best of our knowledge, only four cases have been described in the literature. This uncommon injury is mostly caused by high-velocity impact and associated with life-threatening lesions. We report a unique case of concomitant ipsilateral floating hip and floating knee following road traffic accident. The patient presented ipsilateral hip dislocation and acetabular, femoral, and tibial fractures associated with chest trauma. The aim of this report is to highlight the severity and rarity of this combination and to describe the therapeutic recommendations.

## 1. Introduction

Simultaneous ipsilateral floating hip and floating knee are extremely rare. To the best of our knowledge, only four cases have been published. These special injuries generally occur following high-energy trauma. Hence, they are often associated with life-threatening conditions. In addition, the combination of an ipsilateral floating hip and floating knee remains a surgical challenge in orthopedics. However, there are no sufficient reports regarding the management of such cases. The authors present a unique case of concomitant ipsilateral floating hip and knee injuries, in order to underline the severity of this entity and highlight the importance of early surgical management.

## 2. Case Report

**Figure 1:**
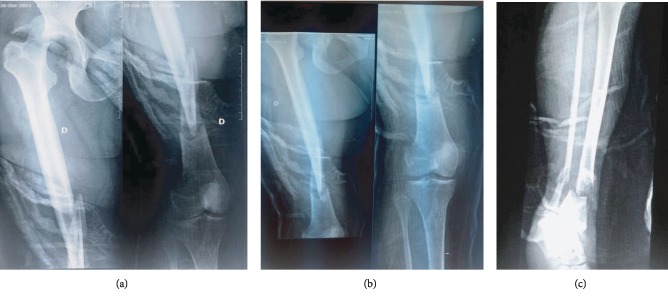
Initial radiographs of the fracture sites. (a, b) Radiographs showing dislocation of the right hip, displaced posterior wall of acetabular fracture, and concomitant femur shaft fracture. (c) Radiograph showing tibia fracture.

**Figure 2:**
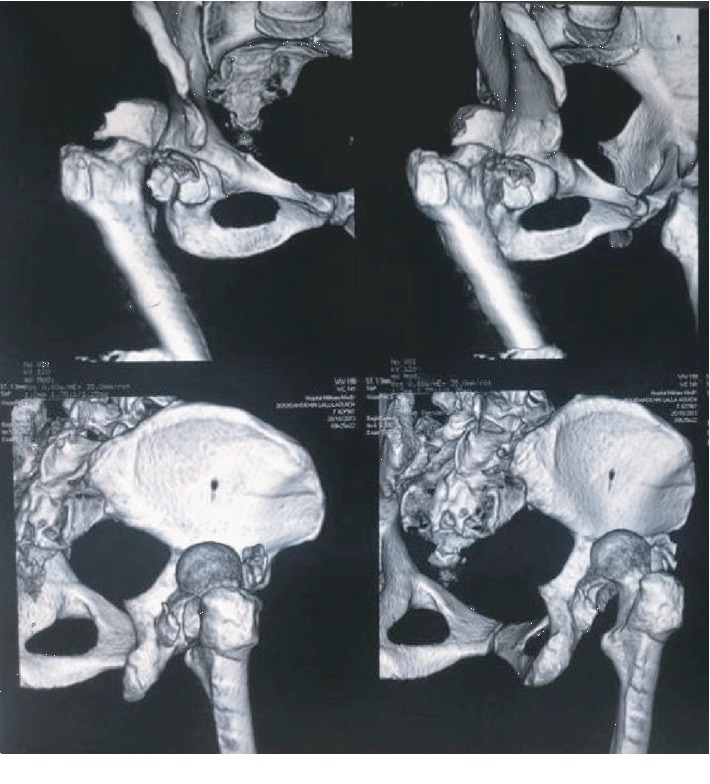
Three-dimensional computed tomography images showing a dislocation fracture of the right acetabulum.

**Figure 3:**
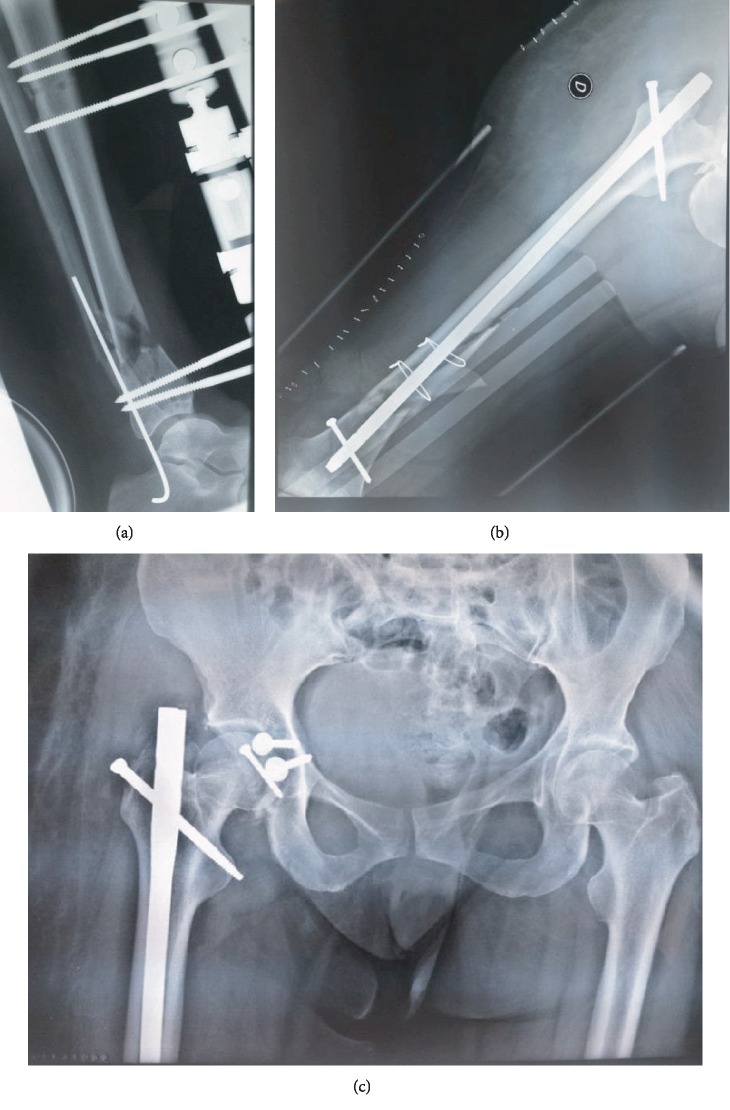
Postoperative radiographs showing fixation of fractures of (a) tibia, (b) shaft of femur, and (c) acetabulum.

A 56-year-old female, who had been followed for diabetes since 2004, was admitted to the emergency service as pedestrian who was hit by a car traveling about 60 miles per hour. On physical examination, she was conscious and hemodynamically stable with a blood pressure of 135/85. Her right lower limb was short and deformed in abduction and external rotation, and there was open wound in the distal 1/3 anterior of the tibia measuring 7 cm with moderate soft tissue injury, corresponding to grade II of the Gustilo classification. The neurovascular examination was uneventful. Radiographs indicated right hip dislocation, displaced posterior acetabular wall fracture (Figure 1(a)), femoral shaft fracture at distal third, and concomitant fracture in the distal third of the tibia (Figures 1(b) and 1(c)). Chest radiograph revealed a small pneumothorax. A pelvis computed tomography confirmed the posterior fracture dislocation and showed the presence of intra-articular fragments (Figure 2). Urgently, the patient was taken to the operation room following resuscitation. Under general anesthesia, an intercostal tube was inserted for the pneumothorax. Closed reduction of the hip dislocation was performed. Then, the open fracture of the tibia was stabilized with an external fixator after debridement of all devitalized tissues (Figure 3(a)).

Two days later, the patient was taken back to the theatre. Under general anesthesia, she was positioned in the left lateral decubitus. First, the diaphyseal femoral fracture was fixed with an antegrade intramedullary nail with 2 distal locking bolts following open reduction (Figure 3(b)). Then, the acetabular fixation was performed through a Kocher-Langenbeck approach. The posterior wall fracture was stabilized using 2 screws due to the presence of large fragment and good bone quality. Finally, the intraoperative exploration found the existence of a detached fragment of the femoral head, which was fixed by direct screw (Figure 3(c)).

**Figure 4:**
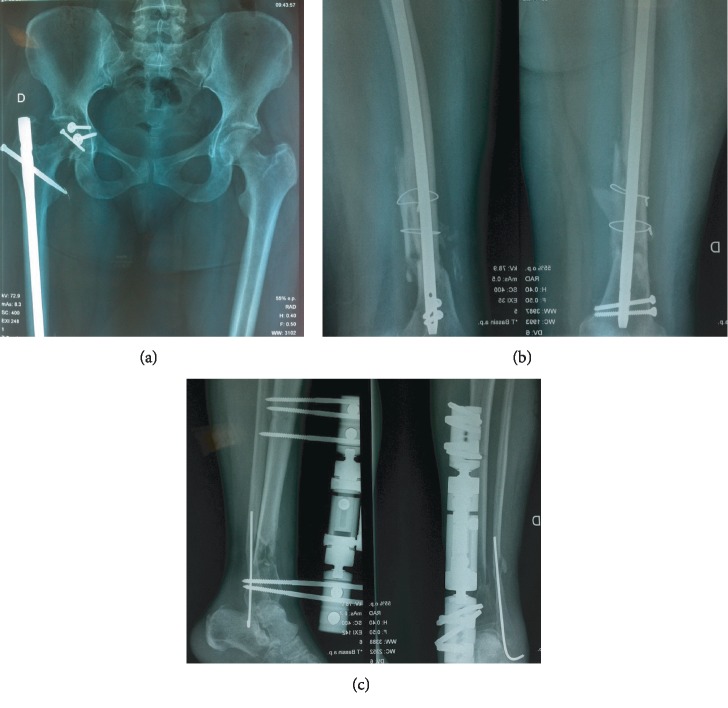
Radiographs of the (a) pelvis, (b) femur, and (c) tibia at 7-month follow-up.

Postoperatively, there was no acute complication such as infection, venous thrombosis. The chest drain was removed on the fifth day. The tibial wound was well cicatrized, and the wound cultures were sterile. After two weeks, the patient was discharged to go home. Initially, she was mobilized non-weight-bearing on crutches for ten weeks. Then, partial weight-bearing was allowed. The rehabilitation protocol included muscular exercises especially quadriceps and early mobilization of the hip and knee for prompt recovery of the joint range of motion. After seven months, the follow-up radiographs showed bony consolidation of all fractures except the tibia which was completely consolidated in 10 months (Figures 4(a)–4(c)). We opted to remove the external fixator. We authorized the full weight-bearing, and we asked the patient to use a walking stick. At 1 year after trauma, the patient returned to normal activities. The hip and knee functions were recovered with limited flexion of the knee at 110 degrees.

## 3. Discussion

Combined ipsilateral fractures above and below articulations are known as floating joints that disconnect the joint from the rest of the limb [1]. Generally, they are indicative of high-velocity trauma especially road traffic accidents as seen in our case. Although floating hip and floating knee are not uncommon, simultaneous floating hip and floating knee on the same limb are very rare combination, and only four reports have been published before [2–4]. The management of such injuries remains a surgical dilemma in orthopedics due to their low prevalence and paucity in literature concerning their management.

Most reports regarding management of the floating hip demonstrated that the surgical fixation of all fractures is the best option with excellent clinical outcome. But, the main treatment dilemma is the optimal sequence of stabilization of these fractures. Indeed, this surgical order has been widely discussed in literature and there are no guidelines concerning which injury will be fixed first. While Liebergall et al. suggested that the femur should be fixed first as this can facilitate reduction and traction of the acetabulum [1], Kregor and Templeman [5] advocated fixing the acetabular fracture at first. On the other hand, Müller et al. [6] reported 42 patients with floating hip and they fixed the femur first in 38% of the cases. In addition, they found a high rate of postoperative complications: iatrogenic injury of the sciatic nerve (24%) and shortening of the femur (21%). Although there is no consensus on management of the floating hip, most authors agree on early stabilization of unstable pelvic injuries as a measure of adequate resuscitation according to the principles of damage control orthopedics [7, 8]. We believe that the surgical order has to be discussed case to case.

In our case, the hip dislocation was urgently treated with a closed reduction. The femoral shaft fracture was fixed first with an antegrade intramedullary nail in the lateral position. Then, the acetabular and femoral head fractures are stabilized at the same time by using the posterior approach. There was no iatrogenic nerve injury, but there was a small shortening of the lower limb than 1 cm.

Floating knee injuries are almost always caused by high-energy trauma. Hence, they are often associated with life-threatening injuries. Rios et al. [9] reported that 42% of patients had head injury, 16% abdominal injuries, and 28% chest trauma. Moreover, it has been reported that the mortality rate of this severe injury range between 5 and 15% [10]. The soft tissues damage is often extensive, and the prevalence of open fractures was approximately 50–70%, especially at the tibia. As early as the first description of floating knee by Blake and McBryde in 1975 [11], various classifications have been reported in the literature and the most commonly used is Fraser's classification. In their report of 222 patients, the authors identified five patterns of floating knee [12].

Currently, immediate surgical fixation of both fractures is generally the treatment of choice with excellent functional results in over 80% patients, but there is no unanimity about the perfect technique of fixation. The latter depends on different parameters: fracture pattern, soft tissue trauma, patient's general condition, and surgeon preference. Several authors [8–10] recommended intramedullary nailing of the femur and the tibia that allows prompt mobilization, permits early weight-bearing, and prevents knee stiffness. It has been reported that the use of retrograde femoral nail permits to stabilize both fractures with a single incision [9, 13] and reduces surgery time with less blood loss. However, the plating is recommended for metaphyseal fractures with articular extension [14]. It is known that internal stabilization has certain benefits over external fixation, but in cases where the soft tissue is significantly involved, external fixation remains the best treatment option. Indeed, Theodoratos et al. advocated internal fixation except for open fractures type IIIB and C [15]. In our case, the floating knee corresponds to type A of the Fraser classification with extra-articular fractures. There was an open fracture in the distal tibia, corresponding to grade II of the Gustilo classification. The treating surgeons opted not to nail both fractures with the same incision for two reasons: the first is that open tibial fracture was treated with external fixation, the second is that the acetabular and femoral fractures were stabilized at the same time with the same approach.

## 4. Conclusion

A simultaneous ipsilateral acetabular, femoral, and tibial injury is a very uncommon combination that is often associated with other vital organ lesions. It seems that aggressive and immediate fixation of all fractures is the current recommendation for these injuries. But this orthopedic management should be performed after stabilization and resuscitation of the polytraumatized patient.
